# Overt and Latent Cardiac Effects of Ozone Inhalation in Rats: Evidence for Autonomic Modulation and Increased Myocardial Vulnerability

**DOI:** 10.1289/ehp.1104244

**Published:** 2011-12-02

**Authors:** Aimen K. Farraj, Mehdi S. Hazari, Darrell W. Winsett, Anthony Kulukulualani, Alex P. Carll, Najwa Haykal-Coates, Christina M. Lamb, Edwin Lappi, Dock Terrell, Wayne E. Cascio, Daniel L. Costa

**Affiliations:** 1 Environmental Public Health Division, National Health and Environmental Effects Research Laboratory, U.S. Environmental Protection Agency, Research Triangle Park, North Carolina, USA; 2 Environmental Sciences and Engineering, University of North Carolina–Chapel Hill, Chapel Hill, North Carolina, USA; 3 Biostatistics and Bioinformatics Research Core Unit, National Health and Environmental Effects Research Laboratory, U.S. Environmental Protection Agency, Research Triangle Park, North Carolina, USA; 4 Curriculum in Toxicology, University of North Carolina–Chapel Hill, Chapel Hill, North Carolina, USA; 5 Office of Research and Development, U.S. Environmental Protection Agency, Research Triangle Park, North Carolina, USA

**Keywords:** air pollution, arrhythmia, autonomic, cardiac, electrocardiogram, heart rate variability, inhalation, latent, overt, ozone, rats

## Abstract

Background: Ozone (O_3_) is a well-documented respiratory oxidant, but increasing epidemiological evidence points to extrapulmonary effects, including positive associations between ambient O_3_ concentrations and cardiovascular morbidity and mortality.

Objective: With preliminary reports linking O_3_ exposure with changes in heart rate (HR), we investigated the hypothesis that a single inhalation exposure to O_3_ will cause concentration-dependent autonomic modulation of cardiac function in rats.

Methods: Rats implanted with telemeters to monitor HR and cardiac electrophysiology [electrocardiography (ECG)] were exposed once by whole-body inhalation for 4 hr to 0.2 or 0.8 ppm O_3_ or filtered air. A separate cohort was tested for vulnerability to aconitine-induced arrhythmia 24 hr after exposure.

Results: Exposure to 0.8 ppm O_3_ caused bradycardia, PR prolongation, ST depression, and substantial increases in atrial premature beats, sinoatrial block, and atrioventricular block, accompanied by concurrent increases in several HR variability parameters that were suggestive of increased parasympathetic tone. Low-O_3_ exposure failed to elicit any overt changes in autonomic tone, heart rhythm, or ECG. However, both 0.2 and 0.8 ppm O_3_ increased sensitivity to aconitine-induced arrhythmia formation, suggesting a latent O_3_-induced alteration in myocardial excitability.

Conclusions: O_3_ exposure causes several alterations in cardiac electrophysiology that are likely mediated by modulation of autonomic input to the heart. Moreover, exposure to low O_3_ concentrations may cause subclinical effects that manifest only when triggered by a stressor, suggesting that the adverse health effects of ambient levels of air pollutants may be insidious and potentially underestimated.

Ozone (O_3_) is a major smog-associated oxidant with well-established respiratory effects, including decrements in lung function, airway injury and inflammation, compromised host defense, and asthma exacerbation (Hollingsworth et al. 2007; Mudway and Kelly 2000). Although the lung has understandably been the target organ of interest, recent epidemiological evidence suggests a positive association between inhaled O_3_ and clinical cardiovascular events linked to coronary artery disease, myocardial infarction, and atherosclerosis (Srebot et al. 2009); these effects are largely independent of exposure to other pollutants. In controlled human exposure studies, O_3_ exposure has reduced maximal oxygen uptake (Gong et al. 1998) and, in combination with ambient particulate matter (PM), increased diastolic blood pressure (Fakhri et al. 2009) and caused arterial vasoconstriction (Brook et al. 2002). Adverse cardiovascular effects, including increased atherosclerotic plaque size (Chuang et al. 2009) and enhanced sensitivity to ischemic injury (Perepu et al. 2010), have also been reported in animal models.

Upon inhalation, O_3_ is thought to oxidate or peroxidate biological molecules (directly or indirectly) at the surface of the respiratory tract, triggering a pathological cascade characterized by lipid peroxidation, enzyme inactivation, free radical formation, altered membrane permeability, and inflammation (Mustafa 1990). Less is known, however, about the mechanisms mediating O_3_-induced cardiovascular responses and the potential influence of O_3_-induced respiratory effects on cardiovascular function. Although preliminary, the available evidence implicates the following mechanisms: vascular oxidative stress, endothelial/vascular dysfunction, inflammation, and altered autonomic tone (Srebot et al. 2009).

Because cardiac impulse formation, propagation, and arrhythmia often result from the modulation of autonomic balance, one of the most conspicuous data gaps in the impact of O_3_ exposure on normal cardiac electrophysiology and heart rate (HR) is the potential contribution of O_3_-induced modulation of autonomic tone to these effects. Additionally, O_3_ exposure at ambient concentrations may not cause overt functional effects, but rather may produce latent or subclinical effects that appear only when the myocardium or specialized conduction system is further stressed, for example, as a result of cellular calcium loading with aconitine. It is uncertain whether O_3_ exposure elicits such effects. We have previously shown that exposure to PM (Carll et al. 2011; Farraj et al. 2009, 2011; Hazari et al. 2009), diesel exhaust (Hazari et al. 2011), or the irritant acrolein (Hazari et al. 2009) in hypertensive or heart failure rats causes functional cardiac effects, including bradycardia, arrhythmia, increased parasympathetic tone, and/or increased sensitivity to triggered cardiac arrhythmia. The purpose of this study was to examine the concentration-dependent effects of acute O_3_ exposure on HR, heart rhythm, HR variability (HRV; a measure of autonomic tone to the heart), electrocardiography (ECG), and pulmonary and systemic inflammation. In addition, we assessed whether O_3_ exposure increases latent vulnerability to cardiac arrhythmia, hypothesizing that O_3_ acts through the autonomic nervous system to prime the heart to react to secondary challenges.

## Materials and Methods

*Animals.* Twelve-week-old male spontaneously hypertensive (SH) rats were obtained from Charles River Laboratory (Raleigh, NC). SH rats were selected because we previously determined (Farraj et al. 2009) that they are more sensitive to the inflammatory and proarrhythmic effects of acute air pollutant exposure [SH rats have higher mean arterial pressure (~ 40 mmHg difference), on average, than do control rats with normal blood pressure at 12 weeks of age (El-Mas and Abdel-Rahman 2005)]. Rats were housed in plastic cages (one per cage), maintained on a 12/12-hr light/dark cycle at approximately 22°C and 50% relative humidity in our Association for Assessment and Accreditation of Laboratory Animal Care–approved facility, and held for a minimum of 1 week before implantation. All protocols were approved by the Institutional Animal Care and Use Committee of the U.S. Environmental Protection Agency (EPA). Rat food (Prolab RMH 3000; PMI Nutrition International, St. Louis, MO) and water were provided *ad libitum*. All rats were randomized by weight. Animals were treated humanely and with regard for alleviation of suffering.

*Experimental design and O_3_ exposure.* SH rats were surgically implanted with ECG biopotential telemeters and then exposed via whole-body inhalation to 0.2 or 0.8 ppm O_3_ or filtered air once for 4 hr. ECG, HR, body temperature, and activity were monitored before, during, and after exposure to O_3_ or air. All telemetered rats were sacrificed 1 day after exposure to O_3_ or air. A second cohort of rats (untelemetered) in each exposure group was sacrificed 1 hr after exposure to assess potential immediate inflammatory or toxicity responses. A third cohort of rats in each exposure group was challenged with aconitine to assess sensitivity to arrhythmogenic challenge. O_3_ was generated by passing extra dry oxygen past an arcing transformer in a model V5-0 ozone generator (Ozone Research & Equipment Corp., Phoenix, AZ). The chamber concentrations (0.2 and 0.8 ppm) were controlled by the computer program DASYLab (version 9.0; DasyTec USA, Amherst, NH), which controlled the opening and closing of a mass flow controller at each chamber. The actual concentration was then read by an O_3_ analyzer (model 400; Teledyne-Advanced Pollution Instruments, Inc., Thousand Oaks, CA), which fed a signal to a proportional, integral, derivative loop control, which then either opened or closed the mass flow controller to maintain the O_3_ concentration in the chamber at the desired level. Rats were acclimated to the whole-body chamber for 1 hr/day for 2 days before exposure to O_3_ or filtered air.

*Surgical implantation of telemeters.* Animals (*n* = 6/group) were anesthetized with an intraperitoneal (ip) injection of 1 mL/kg of 80 mg/mL ketamine hydrochloride, 12 mg/mL xylazine hydrochloride solution (Sigma Chemical Co., St. Louis, MO). The anesthetized rats were implanted with a biopotential radiotelemetry transmitter (model TA11CTA-F40; Data Sciences International, Inc., St. Paul, MN) using aseptic surgical procedures as previously described (Farraj et al. 2009) to obtain an ECG signal similar to that derived from lead II from the standard ECG and to allow measurement of core body temperature. The animals were allowed 2 weeks for recovery from surgery before exposure to O_3_ or air.

*Radiotelemetry data acquisition and analysis.* Radiotelemetry allowed continuous monitoring and collection of ECG data (acquired using Data ART3.01 acquisition software; Data Sciences International, Inc., St. Paul, MN) in unanesthetized rats from the time of implantation of the transmitters until sacrifice. Receivers (model RPC-1; Data Sciences International, Inc.) were positioned underneath home cages or in whole-body exposure chambers during exposure. Sixty-second segments of ECG waveforms were acquired from animals in their home cages and saved at 15-min intervals from the time of surgical recovery through euthanasia. Values were obtained sequentially by animal and represent averages of 60 sec of data per animal for each 15-min period. HR was automatically obtained from the ECG waveform with data acquisition software. Preexposure data permitted each animal to serve as its own control, and animals exposed to air provided time-paired control data. Preexposure baseline data were obtained while the rats were in the whole-body chamber just before the beginning of exposure, collected in 120-sec periods once every 5 min for 1 hr. Whole-body inhalation exposure data were collected in 120-sec periods once every 5 min for the duration of the 4-hr exposure period. The rats were then returned to their home cages, and postexposure data (60 sec of data every 15 min) were collected until euthanasia, approximately 18 hr after the end of exposure.

ECG, arrhythmia identification, and HRV ecgAUTO software (version 2.5.1.35; EMKA Technologies USA, Falls Church, VA) was used for automated analysis of ECG wave amplitudes and segment durations and areas, as well as visual identification and enumeration of cardiac arrhythmias, and arrhythmia analysis. The following parameters were determined for each ECG waveform: PR interval; QRS duration, amplitude, and area; QT interval; HR-corrected QT interval (QTc; Bazett’s formula); ST interval, amplitude, and area; R-wave amplitude and interval; and T-wave amplitude and area. To account for potential effects of normal circadian rhythm, ECG parameters were quantified over four 6-hr periods for time-matched comparisons between preexposure and postexposure periods while the rats were unrestrained in their home cages. The times analyzed were 0000 hours to 0600 hours, 0600 hours to 1200 hours, 1200 hours to 1800 hours, and 1800 hours to 0000 hours. ECG parameters during exposure were analyzed as baseline (120 sec of data collected every 5 min for 1 hr while in the whole-body chamber immediately before the beginning of exposure) and hours 0–4 during exposure (120 sec of data collected every 5 min for 4 hr while in the whole-body chamber during exposure constituting the entire exposure period between ~ 0830 hours and 1230 hours).

Cardiac arrhythmic events were identified in part by using the Lambeth conventions (Walker et al. 1988) as a guideline for the identification of arrhythmias in rats. Arrhythmias were identified as atrial premature beats (APBs), ventricular premature beats, sinoatrial blocks (SABs), atrioventricular blocks (AVBs), or ventricular tachycardia. Arrhythmias were quantified and totaled over an 18-hr period before exposure (this corresponded to the same times assessed after exposure), during the 4-hr exposure period, and during the 18-hr period beginning immediately after exposure. Total arrhythmia counts during exposure were quantified (in a total of 48 two-minute segments during the 4-hr exposure period). To arrive at counts per hour, the total amount of time sampled in minutes (96) was divided by the number of minutes per hour (60).

HRV is the degree of difference in the interbeat intervals of successive heartbeats and is an indicator of the balance between the sympathetic and parasympathetic arms of the autonomic nervous system (Rowan et al. 2007). Low HRV, reflecting increased sympathetic tone (Rowan et al. 2007), is associated with increased cardiovascular morbidity and mortality (Bigger et al. 1993; Corey et al. 2006). For HRV analysis, thorough visual inspection was conducted to identify and exclude arrhythmias, artifacts, and sample periods with < 30 sec of distinguishable R-waves. HRV analysis generated HR and time-domain measures, including mean time between adjacent QRS-complex peaks (RR interval), standard deviation of the time between normal-to-normal (RR) beats (SDNN), SDNN normalized for the effects of HR [SDNN/(RR interval × 100)], root mean square of successive differences in adjacent RR intervals (RMSSD), and percentage of adjacent normal RR intervals differing by ≥ 15 msec (pNN15). pNN15 is a measure of parasympathetic tone comparable to pNN50 in humans. SDNN represents overall HRV, whereas RMSSD represents parasympathetic influence over HR (Rowan et al. 2007). HRV analysis also calculated frequency domain parameters, particularly low frequency (LF), high frequency (HF), and the ratio of these two frequency domains (LF:HF). LF is generally believed to represent a combination of sympathetic and parasympathetic tone, whereas HF indicates parasympathetic tone, and LF:HF serves as an index of sympathovagal balance (Rowan et al. 2007).

*Necropsy, blood collection, and lung lavage.* Rats were deeply anesthetized with an ip injection of Euthasol (200 mg/kg sodium pentobarbital, 25 mg/kg phenytoin; Virbac Animal Health, Ft. Worth, TX) approximately 1 or 18 hr after the end of exposure. Blood samples were collected from the abdominal aorta. The trachea was cannulated, and the right lung (except for the caudal lobe) was lavaged with a total volume of 20 mL/kg Ca^2+^/Mg^2+^/phenol red–free Dulbecco’s phosphate-buffered saline (SAFC Biosciences, Lenexa, MD) divided into two equal aliquots. The caudal lobe was collected for RNA analysis. Cytospins and cells differentials on lavaged cell samples (neutrophils, lymphocytes, macrophages, and eosinophils per millimeter of bronchoalveolar lavage fluid) and assays for total protein (Thermo Fisher Diagnostics, Rockford, IL); albumin (DiaSorin, Stillwater, MN); lactate dehydrogenase (Thermo DMA, Louisville, CO); *N*-acetyl-β-d-glucosaminidase (Roche Diagnostics, Mannheim, Germany); superoxide dismutase (Randox Laboratories Ltd., Crumlin, CO); glutathione peroxidase and glutathione *S*-transferase [based on an in-house automated analysis (Jaskot et al. 1983)], serum C-reactive protein (DiaSorin); creatine kinase (Fisher Diagnostics, Middletown, VA); sorbitol dehydrogenase (Sekisui Diagnostics, Charlottetown, Canada); creatinine (Sekisui Diagnostics); high-density lipoprotein cholesterol (HDL), low-density lipoprotein (LDL) cholesterol, and plasma angiotensin-converting enzyme (Fisher Diagnostics); and fibrinogen (DiaSorin) in lavage supernatants were conducted as previously described (Farraj et al. 2009).

Hearts were weighed and normalized by body weight before necropsy to examine effects of exposure on heart mass.

*Aconitine challenge.* Eighteen hours after exposure to O_3_, a separate cohort of animals were anesthetized with urethane (1.5 g/kg, ip; Sigma Chemical Co.) and underwent the aconitine challenge; supplemental doses of the anesthetic were administered intravenously (i.v.) when necessary to abolish pain reflex. Animal body temperature was maintained at approximately 36°C with a heating pad. The left jugular vein was cannulated with PE-50 polyethylene tubing for the administration of aconitine. Aconitine (10 μg/mL) was continuously infused at a speed of 0.2 mL/min, and ECG activity was continuously monitored and timed. Sensitivity to arrhythmia was measured as the threshold dose of aconitine required to produce ventricular premature beats, ventricular tachycardia, ventricular fibrillation, and cardiac arrest:

Threshold dose (μg/kg) for arrhythmia =10 μg/mL × 0.2 mL/min × time required for inducing arrhythmia (min)÷ body weight (kg) [1]

*Statistics.* The statistical analyses for all data in this study were performed using SAS software (version 9.2; SAS Institute Inc., Cary, NC). PROC MIXED procedure was used to analyze the ECG, HR, and HRV data. A linear mixed model with restricted maximum-likelihood estimation analysis (SAS) and least squares means post hoc test were used to determine statistical differences for all data. All the biochemical and cell differential data were analyzed using analysis of variance (ANOVA) examining the main effects of each model as well as the interactive effects. *p*-Values < 0.05 were considered statistically significant. Pairwise comparisons were performed as an ANOVA subtest, adjusting the significance level for multiple comparisons using Tukey’s post hoc test. A correlation analysis between pairs of variables during exposure was carried out using the Pearson product-moment correlation coefficient (*r*).

## Results

**Figure 1:**
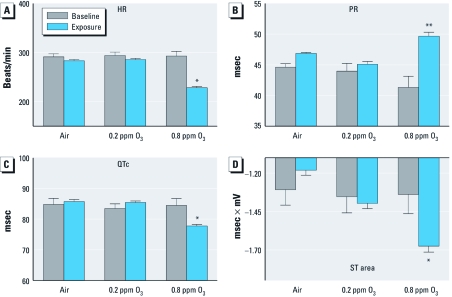
HR (*A*), PR interval (*B*), QTc (*C*), and ST area (*D*) immediately before (baseline) and during (exposure) 4-hr exposure to 0.2 ppm or 0.8 ppm O_3_ or filtered air (mean ± SE, *n* = 6). *Significantly less than preexposure baseline values within corresponding group (*p* < 0.05). **Significantly greater than preexposure baseline values within corresponding group (*p* < 0.05).

*HR and ECG morphology.* High-O_3_ exposure caused a significant decrease in HR (22.1%; *p* < 0.05) relative to preexposure baseline values (Figure 1). There was no significant effect of low-O_3_ or air exposure on HR.

**Figure 2:**
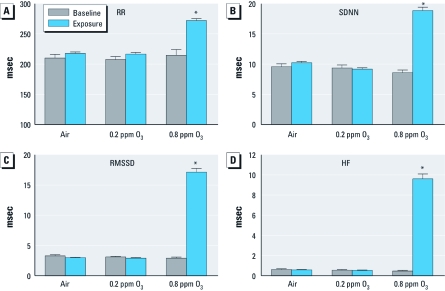
RR interval (*A*), SDNN (*B*), RMSSD (*C*), and HF HRV domain (*D*) immediately before (baseline) and during (exposure) 4-hr exposure to 0.2 ppm or 0.8 ppm O_3_ or filtered air (mean ± SE, *n* = 6). *Significantly greater than preexposure baseline values within corresponding group (*p* < 0.05).

Also relative to corresponding preexposure baseline values, high-O_3_ exposure caused a significant increase in PR interval (20.3%; *p* < 0.05), a significant decrease in QTc (7.9%; *p* < 0.05), and a significant increase in negative ST area (25%; *p* < 0.05) (Figure 1). High-O_3_ exposure also caused a significant increase in RR interval (26.7%; *p* < 0.05; Figure 2). There were no significant effects in any of these parameters in the low-O_3_ or air-exposed groups.

There were no significant postexposure effects in any ECG interval and contour parameters in any groups (data not shown).

*Arrhythmia.* High-O_3_ exposure caused a significant increase in the number of APBs (2,200%; *p* < 0.05), SABs (32,600%; *p* < 0.05), and Mobitz type I second-degree AVBs (1,300%; *p* < 0.05) during exposure relative to preexposure baseline values (Table 1). There was no significant effect of low-O_3_ or air exposure on any measured arrhythmia. There were little to no significant postexposure effects in arrhythmia number in any groups (data not shown).

**Table 1:** Number of arrhythmias per hour immediately before and during the 4-hr exposure period (mean ± SE).

Exposure	Arrhythmia										
	APB			SAB			AVB		
	Baseline	Exposure	Baseline	Exposure	Baseline	Exposure
Air		0.5 ± 0.8		0.2 ± 0.2		1 ± 1		0.1 ± 0.2		0 ± 0		0.2 ± 0.2
0.2 ppm O_3_		3.5 ± 3.5		1.1 ± 0.4		1 ± 1		0.2 ± 0.3		0 ± 0		0 ± 0
0.8 ppm O_3_		1.0 ± 1.0		23 ± 14*		1 ± 1		327 ± 99*		0 ± 0		14 ± 7*
*Significantly greater than corresponding preexposure baseline value (*p* < 0.05).												

*HRV parameters.* High-O_3_ exposure caused a significant increase in SDNN (119%; *p* < 0.05; Figure 2), RMSSD (485%; *p* < 0.05; Figure 2), LF (7,070%; *p* < 0.05; data not shown), HF (1,900%; *p* < 0.05; Figure 2), and LF:HF (137%; *p* < 0.05; data not shown) relative to preexposure baseline values. There was no significant effect of low-O_3_ or air exposure on any measured HRV parameter. There were no significant postexposure effects on HR or any HRV parameters in any of the exposure groups (data not shown).

**Figure 3:**
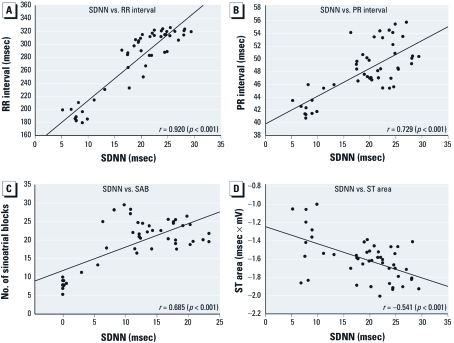
Pearson product-moment correlation analysis between SDNN and RR (*A*), PR interval (*B*), SAB (*C*), and ST area (*D*) during exposure to 0.8 ppm O_3_. Simple regression lines are superimposed on each figure. The Pearson product-moment correlation coefficient (*r*) is indicated for each relationship.

There were significant correlations between high-O_3_–induced increases in SDNN and several time-matched ECG parameters and arrhythmia (Figure 3). SDNN positively correlated with RR (*r* = 0.920; *p* < 0.001; Figure 3) and PR (*r* = 0.729; *p* < 0.001; Figure 3) intervals and SAB (*r* = 0.685; *p* < 0.001; Figure 3) and negatively correlated with ST area (*r* = –0.541; *p* < 0.001; Figure 3) and QTc (*r* = –0.806; *p* < 0.001; data not shown). A correlation between SDNN and APB barely fell below the threshold of significance (*r* = 0.280; *p* = 0.054; data not shown). SDNN significantly correlated with PR prolongation with exposure to low O_3_ (*r* = –0.326; *p* < 0.001; data not shown) and air (*r* = 0.404; *p* < 0.001; data not shown). There were no other significant correlations with SDNN in any of the remaining parameters in the low-O_3_–exposed and air-exposed groups (data not shown).

*Temperature and heart weight.* High-O_3_ exposure caused a significant decrease in core body temperature [see Supplemental Material, Table 1 (http://dx.doi.org/10.1289/ehp.1104244)]. There were no significant effects of low O_3_ or air on core body temperature. There were no significant effects of exposure on heart weight (data not shown).

*Indicators of inflammation in lung and serum.* With few exceptions, O_3_ exposure at both concentrations had no statistically significant effect on indicators of inflammation and injury in lung lavage, serum, and plasma at either 1 or 18 hr after O_3_ exposure. High O_3_ did cause a significant decrease in serum HDL (mean ± SE: air, 34.9 ± 7.2 mg/dL; 0.8 ppm O_3_, 17.2 ± 0.7 mg/dL; 51%; *p* < 0.05) and creatinine (air, 0.62 ± 0.03 mg/dL; 0.8 ppm O_3_, 0.43 ± 0.03 mg/dL; 31%; *p* < 0.05) and a significant increase in serum sorbitol dehydrogenase (air, 14.1 ± 2.6 U/L; 0.8 ppm O_3_, 30.9 ± 6.4 U/L; 119%; *p* < 0.05) relative to air controls 24 hr after exposure. High O_3_ caused a small increase (62% relative to air group) in lung lavage neutrophils 18 hr after exposure that was not statistically significant [Supplemental Materials, Table 2 (http://dx.doi.org/10.1289/ehp.1104244)].

**Figure 4:**
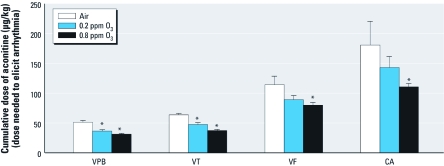
Cumulative dose of infused aconitine necessary to trigger ventricular premature beats (VPB), ventricular tachycardia (VT), ventricular fibrillation (VF), and cardiac arrest (CA) in rats approximately 18 hr after a single exposure to 0.2 ppm or 0.8 ppm O_3_ or filtered air (mean ± SE, *n* = 5). *Significantly less than air control for corresponding arrhythmia (*p* < 0.05).

*Sensitivity to aconitine.* Eighteen hours after O_3_ exposure, both low- and high-O_3_ exposure significantly reduced the total dose of aconitine necessary to elicit the first ventricular premature beat relative to air-exposed controls (28% and 39%, respectively; *p* < 0.05; Figure 4). Both low and high O_3_ also significantly reduced the total dose of aconitine necessary to elicit the first episode of ventricular tachycardia relative to air-exposed controls (26% and 42%, respectively; *p* < 0.05). Only the high O_3_ concentration significantly reduced the total dose of aconitine necessary to elicit the first episode of ventricular fibrillation and cardiac arrest relative to air-exposed controls (30% and 39%, respectively; *p* < 0.05).

## Discussion

We found that inhalation of O_3_ for a brief period causes concentration-dependent overt and latent neurophysiological and electrophysiological effects in SH rats, a strain of rat known to demonstrate cardiac effects in response to inhaled ambient PM, diesel exhaust particles, and acrolein (Farraj et al. 2009, 2011; Hazari et al. 2011). Among the most striking effects with high (0.8 ppm) but not low (0.2 ppm) O_3_ were ECG alterations that were suggestive of changes in repolarization (ST depression and QT shortening) and atrioventricular conduction block (PR prolongation). Only the high-O_3_ exposure caused significant ST depression. Although rats lack an equivalent human ST segment because of the rapidity with which their ventricular myocytes repolarize, perturbations that result in ST segment changes in species with ST segments produce a similar shift in the corresponding QRS-T wave region of the ECG in rats (Detweiler 1981). ST segment depression in humans is temporally associated with myocardial ischemia (Detweiler 1981) and has been associated with exposure to other air pollutants, including PM (Pekkanen et al. 2002). Although the ST segment changes are suggestive of ischemia, measurements of biological indicators of ischemia were not performed in this study and will be needed to confirm ischemia in future studies. High-O_3_ exposure also caused QT shortening, but the significance of this finding is unclear. Moreover, high-O_3_ exposure prolonged the PR interval, providing evidence of slowing between atrial and ventricular activation. Uchiyama et al. (1986) reported similar findings with 1 ppm O_3_ in rats. PR prolongation is usually associated with increased parasympathetic tone (Sapire et al. 1979).

High- but not low-O_3_ exposure increased episodes of APBs, SAB, and second-degree Mobitz type I AVB arrhythmias, consistent with the proarrhythmic effects of O_3_ described in several epidemiological studies (e.g., Chiu and Yang 2009; Sarnat et al. 2006). APBs are ectopic beats that originate within the atria and have been linked to increased parasympathetic tone (Wilhelm et al. 2011). These findings are consistent with those reported by Uchiyama et al. (1986), where O_3_ exposure caused increased APBs and AVBs in rats. Furthermore, this is the first study to report an increase in SAB with O_3_ exposure in an experimental model. SAB in humans is believed to be caused by a block of conduction within the sinoatrial junction while the sinus node itself functions normally (Chung 1983). High-O_3_ exposure also increased AVB, which in humans is characterized by the failure of some atrial impulses to be conducted to the ventricles (Chung 1983). Both SAB and AVB have also been linked with increased parasympathetic tone (Page et al. 1991). The exact sites of the blocks produced in the present study are not known and could have been obtained with only intracardiac recordings, which were beyond the scope of this study. Although it is unclear whether such anatomical lesions exist in rats and what, if any, translational clinical significance they have, these findings point to an increased proclivity to the development of arrhythmias with O_3_ exposure. The predisposition of susceptible individuals to the development of cardiac arrhythmias after O_3_ exposure and the mechanisms mediating these responses need to be further studied.

Only rats exposed to high O_3_ had a significant decrease in HR and a significant increase in multiple HRV parameters, including SDNN, RMSSD, and HF, all of which indicate a shift toward increased parasympathetic tone. High-O_3_ exposure also caused an increase in LF and LF:HF. LF, however, is a poor indicator of sympathetic tone, particularly in heart failure patients (Notarius et al. 1999), and instead may reflect an interaction of the sympathetic and parasympathetic nervous systems (Houle and Billman 1999). Multiple reports using experimental models indicate similar decreases in HR with O_3_ exposure (e.g., Uchiyama et al. 1986; Watkinson et al. 2001). Moreover, Peel et al. (2011) found that O_3_ exposure is associated with increased occurrence of apnea and bradycardia in high-risk infants, suggesting similarly elevated parasympathetic tone, and Davoodi et al. (2010) demonstrated that air-pollution–induced increases in arrhythmias were linked with increased RMSSD. Analogous findings have been reported in experimental models, including cholinesterase inhibition after O_3_ exposure in guinea pigs (exaggerates parasympathetic activity; Gordon et al. 1981), reversal of O_3_-induced bradycardia with atropine in rats (parasympathetic blocker; Arito et al. 1992), and vagotomy in dogs (Vaughan et al. 1971). These findings contrast with recent epidemiological studies pointing to decreased HRV and increased sympathetic tone with O_3_ exposure (Schwartz et al. 2005; Zanobetti et al. 2010). The disparity in effects may be explained by the timing of assessments because, as we have previously demonstrated, rats transition from elevated parasympathetic tone during exposure to air pollutants (Farraj et al. 2011) to sympathetic mediation of cardiac effects 1 day after exposure (Hazari et al. 2011). Nevertheless, increased HRV may also have links to adverse health outcomes. For example, Farkas et al. (2008) showed that increased parasympathetic tone is a precursor to drug-induced torsade de pointes (a precursor arrhythmia to ventricular fibrillation; Gralinski 2003) and is associated with increased apnea severity in obese patients (Reynolds et al. 2007), adverse cardiovascular events in type II diabetics (Eguchi et al. 2010), and increased mortality in heart failure (Stein et al. 2005). The relationship between increased parasympathetic tone, O_3_ exposure, and cardiac dysfunction requires further study.

The concurrence of HRV, ECG, and arrhythmia changes during exposure coupled with their strongly significant correlation suggests a potential interdependence. O_3_-exposure–induced increases in SDNN positively correlated with PR prolongation and increased SAB and negatively correlated with HR, QTc, and ST area. Although not proving a direct cause–effect relationship, these findings suggest that increased parasympathetic tone may have played a role in the contemporaneous induction of several ECG anomalies. The rapid onset of these responses suggests triggering by sensory irritation originating in the nose or lung. The activation of irritant nerve fibers, including pulmonary C-fibers, by air pollutants elicits a reflex cardiopulmonary response characterized by apnea, bronchospasm, hypotension, and bradycardia (Widdicombe and Lee 2001). Although Jimba et al. (1995) showed that O_3_ does not activate transient receptor potential (TRP) channel V1-expressing C-fibers, Taylor-Clark and Undem (2010) recently demonstrated that O_3_ exposure activates TRPA1-expressing airway C-fibers. Moreover, our group has shown that increases in sensitivity to aconitine-induced arrhythmia after diesel exhaust exposure is dependent on the activation of TRPA1 on airway sensory nerves (Hazari et al. 2011). Thus, the enhanced sensitivity to O_3_ in this study may also have been driven by activation of the TRPA1 receptor; future studies will be needed to confirm this.

The impact of the small changes in lung neutrophils, serum HDL cholesterol, creatinine, and sorbitol dehydrogenase on the observed cardiac responses is unclear. The absence of significant cellular inflammation and oxidative changes, however, suggests that these phenomena played little to no role in the elicitation of these cardiac responses. High O_3_ also caused a 3°C drop in core body temperature during exposure; such drops in body temperature are believed to be part of a hypothermic response to toxicants unique to rodents that serves to protect the animal from further injury (Gordon et al. 1988; Watkinson et al. 1997). Bradycardia, bradyarrhythmias, and PR prolongation have all, however, been associated with reduced internal body temperature in humans, particularly in hypothermia (de Souza et al. 2007). Further work is required to determine whether temperature changes play any role in eliciting such cardiac effects.

A striking outcome of both high- and low-O_3_ exposure was an increased sensitivity to cardiac arrhythmia triggered 1 day after exposure, indicating latent/indirect consequences of a single exposure to this oxidant air pollutant. Aconitine, a cardiotoxic alkaloid used commonly to induce experimental arrhythmia, suppresses inactivation of tetrodotoxin-sensitive sodium channels in the myocardium and other excitable tissues (Hazari et al. 2009). Increased sensitivity to aconitine suggests that O_3_ exposure altered the degree to which the cardiovascular system can withstand stress by lowering the threshold for the initiation of adverse ventricular arrhythmias. These results are similar to our previous findings with particulate and gaseous pollutants (Hazari et al. 2009, 2011) suggesting that air pollutant exposure increases the sensitivity of the cardiac electrical conduction system in a nonspecific fashion. The exact nature of this alteration is unclear. One plausible possibility is slowing of the ventricular activation rate secondary to increased parasympathetic influence, with attendant intracellular calcium loading (Shattock and Bers 1989) and superimposed increased fast sodium current. These phenomena may have caused sarcoplasmic reticular overload, leading to spontaneous calcium release and triggered activity (a mechanism of arrhythmia formation). Moreover, ventricular remodeling may be a contributing factor because SH rats undergo changes in ion channel expression (Goltz et al. 2007) during progression of their hypertensive phenotype that may heighten myocardial sensitivity. Further work is required to confirm any myocardial changes or other mechanisms that may account for this phenomenon.

Perhaps most compelling is our finding that 0.2 ppm O_3_ also increased sensitivity to aconitine-triggered cardiac arrhythmia despite failing to elicit any direct overt cardiac alterations as were observed with exposure to 0.8 ppm O_3_. These findings indicate that exposure to low O_3_ concentrations may cause subclinical/insidious effects that manifest only when triggered by a stressor, suggesting that the health effects of ambient levels of air pollutants may be insidious and potentially underestimated.

## Conclusion

O_3_ exposure caused HR and ECG changes that were accompanied by a shift in sympathovagal balance, but no apparent significant cellular inflammation, indicating potential mediation by increased parasympathetic tone and less dependence on the overt injury and inflammation common at high concentrations. Sensory/irritant responses (e.g., pulmonary C-fiber activation) may have played a role in triggering these autonomic/ECG effects and should be examined in future studies. Perhaps of greater significance is the finding that O_3_ causes latent effects, suggesting that exposure would render a subject acutely sensitive to the effects of a nonspecific cardiac trigger. This presumably transient window of hypersensitivity is particularly worrisome in individuals with preexisting cardiovascular disease who are already burdened by a reduced capacity for compensation. Thus, the wealth of knowledge we have on the direct effects of O_3_ may not fully inform us of the complex cardiopulmonary response profile of this oxidant. That this latent cardiac effect was present at concentrations that caused no overt toxicity [i.e., 0.2 ppm, approximately three times the U.S. EPA’s current O_3_ 8-hr National Ambient Air Quality Standard of 0.075 ppm (U.S. EPA 2006)] is alarming and suggests that controlled human and experimental exposure studies may underestimate the effects of exposure. Conversely, these findings are consistent with epidemiological studies that demonstrate adverse effects with relatively small spikes in ambient O_3_ concentrations (e.g., 10 ppb; Bell et al. 2004). Collectively, these findings provide new insight into the effects and mechanisms of O_3_ and highlight the complexity of the assessment of the cardiovascular toxicity of different air sheds.

## Supplemental Material

(61 KB) PDF
