# Supplementary material for: Overt and Latent Cardiac Effects of Ozone Inhalation in Rats: Evidence for Autonomic Modulation and Increased Myocardial Vulnerability
Source: Environ Health Perspect. 2011 Dec 2;120(3):348–54. doi: 10.1289/ehp.1104244 (PMC3295357; doi:10.1289/ehp.1104244)
Supplement: (61 KB) PDF [file ehp.1104244.s001.pdf]

Supplemental Material

Overt and Latent Cardiac Effects of Ozone Inhalation in Rats: Evidence for Autonomic Modulation and Increased Myocardial Vulnerability

Aimen K. Farraj[1], Mehdi S. Hazari[1], Darrell W. Winsett[1], Anthony Kulukulualani[1], Alex P. Carll[2], Najwa Haykal-Coates[3], Christina M. Lamb[4], Edwin Lappi[1], Dock Terrell[1], Wayne E. Cascio[1], and Daniel L. Costa[5].

[1]Environmental Public Health Division, NHEERL, US Environmental Protection Agency, Research Triangle Park, NC, [2]Environmental Sciences and Engineering, University of North Carolina, Chapel Hill, NC, [3]Biostatistics and Bioinformatics Research Core Unit, NHEERL, US Environmental Protection Agency, Research Triangle Park, NC, [4]Curriculum in Toxicology, University of North Carolina, Chapel Hill, NC, [5]Office of Research and Development, US Environmental Protection Agency, Research Triangle Park, NC

Corresponding author: Aimen K. Farraj, Ph.D DABT; U.S. Environmental Protection Agency, Environmental Public Health Division, Mail Code: B105-02, Research Triangle Park, NC 27711; e-mail: farraj.aimen@epa.gov; Tel: (919) 541-5027; Fax: (919) 541-0034

Table of Contents

Page 3 **Supplemental Material, Table 1**

Page 3 **Supplemental Material, Table 2**

Supplemental Material, Table 1: Temperature during 4 hr exposure period

Groups	Baseline	Hour 1	Hour 2	Hour 3	Hour 4
Air	38.3±0.03	37.76±0.07	37.39±0.04	37.16±0.01	37.23±0.02
0.2 ppm O_3	38.22±0.04	38.06±0.06	37.50±0.01	37.29±0.03	37.30±0.02
0.8 ppm O_3	38.45±0.06	38.09±0.08	36.19±0.20*	34.81±0.03*	34.70±0.02*

Values represent means immediately before and during 4 hr exposure period ± standard error of the mean; * = significantly less than corresponding pre-exposure baseline value ($p<0.05$).

Supplemental Material, Table 2: Inflammatory cells per ml BAL

Cell Type	Air	0.2 ppm O_3	0.8 ppm O_3
Neutrophils/ml BAL	1.3± 0.0.3 (x 10^4)	1.8± 0.8 (x 10^4)	2.1± 0.4 (x 10^4)
Lymphocytes/ml BAL	2.3± 0.5 (x 10^4)	2.4± 0.8 (x 10^4)	0.9± 0.3 (x 10^4)
Macrophages/ml BAL	38 ± 2.0 (x 10^4)	32 ± 3.0 (x 10^4)	24 ± 3.0 (x 10^4)
Eosinophils/ml BAL	0.1 ± 0.1 (x 10^4)	0.0 ± 0.0 (x 10^4)	0.0 ± 0.0 (x 10^4)

BAL = bronchoalveolar lavage fluid
